# Could Motor Imagery Training Provide a Novel Load Management Solution for Athletes? Recommendations for Sport Medicine and Performance Practitioners

**DOI:** 10.1177/19417381241297161

**Published:** 2024-11-22

**Authors:** Dominic G. McNeil, Riki S. Lindsay, Ryan Worn, Michael Spittle, Tim J. Gabbett

**Affiliations:** † Institute of Health and Wellbeing, Federation University Australia, Victoria, Australia; ‡ Institute of Education, Arts and Community, Federation University Australia, Victoria, Australia; § Institute of Health and Sport, Victoria University, Victoria, Australia; ‖ Health Innovation and Transformation Centre, Federation University, Ballarat, Victoria, Australia; ¶ Gabbett Performance Solutions, Brisbane, Australia

**Keywords:** internal load, mental imagery, training substitute

## Abstract

**Context:**

Athletes often face the dual challenge of high training loads with insufficient time to recover. Equally, in any team, sports medicine and performance staff are required to progress training loads in healthy athletes and avoid prolonged reductions in training load in injured athletes. In both cases, the implementation of a well-established psychological technique known as motor imagery (MI) can be used to counteract adverse training adaptations such as excessive fatigue, reduced capacity, diminished performance, and heightened injury susceptibility.

**Study Design:**

Narrative overview.

**Level of Evidence:**

Level 5.

**Results:**

MI has been shown to enhance performance outcomes in a range of contexts including rehabilitation, skill acquisition, return-to-sport protocols, and strength and conditioning. Specific performance outcomes include reduction of strength loss and muscular atrophy, improved training engagement of injured and/or rehabilitating athletes, promotion of recovery, and development of sport-specific skills/game tactics. To achieve improvements in such outcomes, it is recommended that practitioners consider the following factors when implementing MI: individual skill level (ie, more time may be required for novices to obtain benefits), MI ability (ie, athletes with greater capacity to create vivid and controllable mental images of their performance will likely benefit more from MI training), and the perspective employed (ie, an internal perspective may be more beneficial for increasing neurophysiological activity whereas an external perspective may be better for practicing technique-focused movements).

**Conclusion:**

We provide practical recommendations grounded in established frameworks on how MI can be used to reduce strength loss and fear of reinjury in athletes with acute injury, improve physical qualities in rehabilitating athletes, reduce physical loads in overtrained athletes, and to develop tactical and technical skills in healthy athletes.

For sports scientists and coaches involved in athlete preparation, the goal of training is simple - maximize performance while minimizing injury risk. At the core of this goal is training load, the prescription of an appropriate training stimulus to attain specific performance goals, and adequate recovery to elicit adaptation. There is good evidence that an optimum training load exists, where insufficient or excessive training may both increase the risk of injury and underperformance.^10,11,12^ For many less experienced coaches, the idea of training load is to provide as much training as possible followed by complete rest; however, loading an athlete this way is neither optimal nor effective.^11,12^ Although, at first, the task of appropriate loading may appear simple, the challenge lies in balancing the external load (work performed) and internal load (the psychophysiological cost of that work), with sufficient rest to maximize adaptation.^74^

Sport practitioners need to consider the frequency, intensity, time, type, and volume of exposure, to elicit desired adaptations, which are dependent on the type of training performed.^4,23,50,63^ Simply performing specific training, however, is not sufficient, as the delivery of the training load and how intensity and rest are applied within a week or cycle needs to be considered. Common training intensity distribution models that target the principle of variation include the polarized and pyramidal approaches,^32^ and whereas they differ in time spent in intensity zones, both share a common link, in that more time is spent in easy zones and less time in very hard zones. Yet, there is a finite amount of time an athlete can spend in high-intensity zones before fatigue and potential overtraining issues arise.^31^

The amount of recovery required between loading periods may differ between athletes, especially if differences in fitness and training experience are present. Individuality is critical to athlete development to maximize training adaptations and avoid potentially unwanted training effects such as excessive fatigue, soreness, increased injury risk, and potential psychological (or mood disturbance) effects such as diminished motivation.^73^ Despite the need for individual load management, an accepted rule of load management to improve capacity is that of progressive overload, where an athlete is exposed gradually to loads greater than they are currently experiencing.^13^ For an athlete to improve upon their current fitness, they must be exposed to increasingly challenging stress and appropriate recovery. If the rate of loading is too quick, or the amount of load is too great or too different (ie, the modality) from that in which the athlete is accustomed, the risk of injury is increased.^12^ This highlights the need for an individualized approach with high specificity for a particular athlete and task.

To mitigate potential issues, the athlete must employ a varied training approach, in which difficult training is followed by recovery. The appropriate application of training variation might explain the success of the polarized approach for endurance and sprint athletes.^24,25,70^ Traditionally, periods of low load have been seen as periods of rest with a reduction of physical training. Although reductions in external load are important for supercompensation to occur, these periods may provide an opportunity for mental training that involves low physical load. This mental training could come in the form of motor imagery (MI), defined as a dynamic mental state during which the representation of a given motor act is rehearsed in working memory, without any overt motor output, where an athlete may be able to enhance their performance through mental rehearsal.^9,21^ As MI presents a novel way to potentially enhance performance during periods of lower training load, this paper will explore the potential benefits of MI and discuss considerations for implementation into a training program.

## Understanding MI and How it is Used

The ability to generate imagery that simulates sensations, actions, and other experiences in the absence of a physical stimulus is one of the most incredible capacities of the human mind.^42^ MI is described as the mental creation or recreation of a specific skill or movement in the absence of overt motor execution, involving a multisensory simulated experience including sights, sounds, feelings, or bodily physiological experiences.^33,44^ The benefit of MI is that the image can involve either simple or complex realistic impressions of a previously- experienced scenario or a creation of an experience that may be of an upcoming event such as a training or competition scenario, presenting the imager with a positive or negative experience.^44^ For example, an athlete can deliberately generate a sport-specific scenario of a movement with the image generated offering numerous intentional stimuli (ie, objects, colors, movement pathways of objects, spatial relations between objects, performance outcomes), and the experience presents a realistic event or scenario to the imager generating the image.^45^ Thus, MI represents the unfolding of the motor representation in the absence of overt movement,^9,30^ providing a realistic opportunity to remember, plan for the future, navigate, and make decisions.^55^

Most athletes use some form of imagery as part of their psychological skills training techniques.^17,51^ There is substantial support for MI in improving key learning and performance outcomes relevant to sport settings, such as maximal voluntary strength,^53^ muscle activation during anterior cruciate ligament (ACL) rehabilitation,^54^ reactive decision-making,^45^ and motor skill development.^39^ Although physical practice is more effective than imagery or mental practice, imagery rehearsal is more effective than no practice. However importantly, a combination of MI and physical practice is better than physical practice alone.^37,65,69^ Yet, for imagery to be an effective rehearsal technique, performance characteristics generated in imagery should provide the imager with an opportunity to rehearse realistic components critical to improve task performance.^19,27,46,72^

Understanding approaches to make MI most effective is key for sport practitioners and coaches. Thinking about the structure of imagery design can contribute positively to an athlete’s ability to learn and perform, while considering important cognitions such as self-efficacy, and regulating arousal and anxiety.^64^ One model proposed to support the use of imagery is the Revised Applied Model of Deliberate Imagery Use,^6^ which comprises 9 components that relate to the application of MI in sport, dance, exercise, and rehabilitation. This model aims to provide guidelines for practitioners to appropriately align imagery type and specific situations.^6^ According to the model, an essential task for practitioners is to understand that individual and situational factors can impact the overall outcome of training and impact the overall efficacy of MI.^56^ Thus, the model focuses on where, how, who, why, what, meaning, imagery ability, and outcome associated with imagery use. The scenario being imagined needs to be considered by the athlete, but important components associated with the outcome of performance should also be considered when initiating an imagery intervention. For example, quality imagery should target specific sport performance components, but also include meaningful propositions to the scenario, while also considering the physical characteristics of the performer and event such as fatigue, perspiration, and tension, because these physiological and emotional reactions are usually included in actual performance.

## How Is it Possible to Imagine Training and Improve Actual Performance?

MI research has revealed that neural and functional bases of action representation, perception, and production resemble real-world performance.^1,71^ The parallels between imagery and physical performance have led researchers to describe imagery as an internal simulation of action.^8^ The observed benefits of MI training have frequently been explained through motor simulation theory, proposing that MI and overt motor execution activate similar motor areas of the brain, using a shared mental representation, and are therefore functionally equivalent.^29,30,43^ As Moran et al^42^ suggest, “imagery not only allows people to rehearse what they would do in certain hypothetical situations but also leads to measurable psychophysiological changes associated with the response and meaning propositions triggered by the situation being imagined (p. 185).”

A number of studies have provided support for a functional equivalence explanation for MI, with findings demonstrating significant similarities in motor-related regions of the brain during MI and actual movement.^26,48^ More importantly for practitioners, research has demonstrated that MI training can lead to changes in central neural structures, specifically the corticospinal pathway. MI may facilitate learning and performance by activating similar neural structures as physical movement, which could lead to functional, training-related changes. Therefore, practitioners should consider how they can design and deliver MI training that accurately mimics actual movement to maximize functional equivalence.^27^

## Understanding MI for Training Load Management

MI may offer an alternative approach to complete rest, by actively facilitating increased performance capabilities while mitigating the negative adaptations of over- and undertraining such as excessive fatigue, reduced performance, and increased injury risk. The functional equivalence hypothesis indicates that MI activates similar pathways to physical performance; therefore, MI should not be considered as complete form of rest. One advantage of MI is that athletes can use MI for several functions in sport and exercise, such as skill development, strategies and problem-solving, increased muscular strength, reduced strength loss, and improved rehabilitation outcomes.^20^ Consequently, MI is a versatile cognitive strategy that can allow an athlete to continue training at a reduced physical training load.

How MI is used is often dependent on the requirements of the athletes. For example, previous research has demonstrated that MI can improve performance and learning in a range of sport-specific skills, including tennis service and ground strokes, basketball free throws, and gymnastics routines.^37,65^ In addition, MI increases self-confidence, improves stress coping abilities, and reduces negative psychological symptoms associated with injury (eg, pain perception, fear of reinjury, anxiety).^3,54,56^ With respect to training management, MI can be utilized to effectively manage training load by:

• increasing muscular activity and contribute to the restoration of local tissue capacity, thereby reducing strength loss and muscle atrophy due to lack of neuromuscular activation;
• practicing sport-specific skills and game tactics;
• engaging injured and rehabilitating athletes in training sooner by promoting simulated physical performance;
• promoting recovery and decreasing risk of injury by replacing or augmenting physical training during phases of overtraining; and
• combining with physical training to provide additional training load to enhance the development of physical qualities, local tissue capacity, and sport-specific skills.

**Figure 1.**
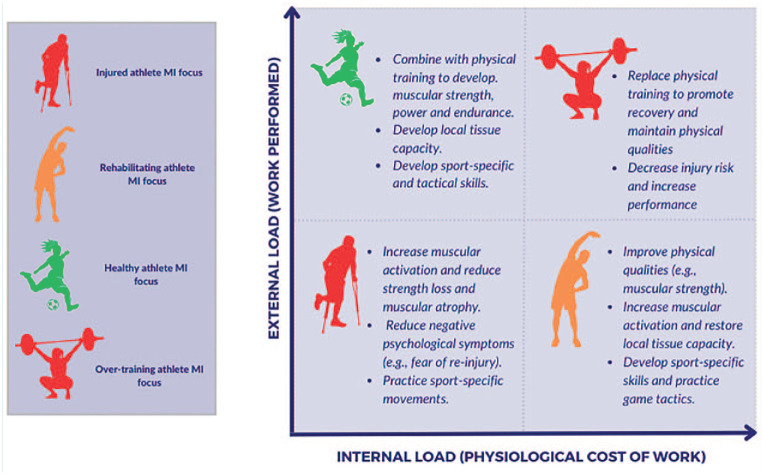
MI use for training load management (adapted from Gabbett et al^15^). MI, motor imagery.

As shown in Figure 1, MI can be used across the training cycle in both healthy and injured athletes to achieve specific performance outcomes. MI can be used when working with healthy athletes to supplement physical training, as an alternative to high training load, substitute for recovering athletes, or promotion of physical capacity to athletes who are limited by injury.

### Mental Lifting: Positive Benefits of MI for Athlete Performance

One specific benefit of MI for training load management is that MI rehearsal has been shown to have positive advantages for promoting key physical capabilities with athletes. Like physical training, load applied progressively using MI training has been shown to elicit training adaptations in key physical capacities, such as muscular strength, power, and endurance.^35,57,66,68,77^ Given the need to balance high training loads with low-intensity training sessions (ie, variation principle), manipulating the type of MI (eg, MI combined with training or MI alone) could effectively moderate the intensity of training without compromising overall performance.

There are periods throughout a training cycle when training load might be reduced to minimize the risk of maladaptation. MI presents a unique opportunity to combat reductions in local tissue and sport-specific capacity associated with prolonged decreases in training (ie, reversibility principle) by “replacing” a portion of physical training with MI training.^57,77^ Findings from Reiser et al^57^ support this notion by demonstrating that different ratios of MI and physical training (75%, 50%, and 25% MI) produced similar increases in maximal voluntary contraction of bench press, leg press, triceps extension, and calf raises relative to physical training. Aligning with the principle of specificity, MI has also demonstrated the potential to produce training-specific muscular responses.^22,75^ Furthermore, research suggests that, despite the MVC of specific muscles through MI, such training does not induce additional neuromuscular fatigue.^60^ However, these findings should be interpreted with caution as others have shown that overall muscular fatigue is increased during MI of muscular endurance-based tasks.^18^ These findings indicate that MI of a specific type of training can be expected to potentially produce appropriate biomechanical responses for that training in the corresponding tissue.

### Training When You Cannot Train: MI for the Rehabilitating Athlete

During periods of injury, global training loads are reduced, followed by a progressive increase in local tissue capacity and neuromuscular control. There is a unique opportunity for cognitive tools such as MI to be utilized during rehabilitation to mitigate the negative adaptations associated with inactivity, such as muscular atrophy and reductions in muscular strength.^40^ The inclusion of MI in the rehabilitation process has been shown to have positive physiological effects that may accelerate recovery, such as increased muscular activation and strength following ACL reconstruction.^35,36,41,49,52,54^ For example, Lebon et al^36^ demonstrated that 12 sessions of MI training alone significantly increased muscular activation of the quadriceps following reconstructive ACL surgery. Such findings suggest that MI could be an effective tool for practitioners to progressively restore local tissue capacity and improve neuromuscular control of the injured limb.

When injured, the decreases in local tissue capacity are driven primarily by tissue damage and pain. However, psychosocial factors, such as fear of reinjury and pain perception, can mediate the effect of injury, often representing a significant barrier to an athlete’s ability to engage in rehabilitation.^2,38^ In the early stages of injury, management of psychological factors is necessary to progress training and facilitate tissue strengthening and neuromuscular control. A clear advantage of MI is that, in addition to the potential neurophysiological benefits already discussed, it may also improve key psychological outcomes that inhibit the rehabilitation process. For example, Rodriguez et al^59^ found that, during ACL rehabilitation, MI significantly reduced athletes’ fear of reinjury and perceptions of pain. Thus, MI could be used to manage psychological factors that prevent an athlete from re-engaging in the training necessary to restore function.

## How Should MI Training Be Structured?

Similar to physical training, MI can be structured systematically by implementing foundational training principles, such as frequency, intensity, type, and time.^67^ Recent systematic and meta-analytic reviews on healthy athletes indicate that the optimal MI frequency is 3 to 4 sessions per week.^37,53,65^ Regarding intensity, MI training is sensitive to changes in training load (i.e., intensity). For example, Guillot et al^22^ showed that the magnitude of electromyographic activity increased and decreased when using MI to perform a bicep curl with more or less effort. These findings indicate that training intensity could be prescribed to mirror loads used during physical movement.

The principle of type refers to the mode of training—in this case, MI alone or combined with physical practice. Previous research indicates that MI combined with physical training is superior to MI alone and, in some cases, more effective than physical training alone.^65^ Therefore, practitioners should consider MI primarily as an addition to physical training schedules, rather than a replacement for physical training. However, the type of MI used will depend on an athlete’s individual context. For the healthy athlete, practitioners may consider adjusting the ratio of MI to physical training as a strategy for managing training load and mitigating negative training adaptations. However, for the rehabilitating athlete, MI alone could be implemented in the early stages of injury to increase local tissue loads through mentally reactivating affected musculature and potentially expediting the rehabilitation process.^47^

It is important to acknowledge that there is an increased cognitive load associated with MI training. Recent research shows that MI performed for >30 minutes can induce mental fatigue, potentially counteracting the positive effects of MI on performance outcomes.^28,61^ Consequently, to appropriately manage the potential impacts of mental fatigue from MI training, practitioners are encouraged to limit MI session duration to <30 minutes.

Finally, beneficial effects of a MI training program have been observed in programs from 1 to 6 weeks in duration, with sessions lasting between 15 and 30 minutes or comprising 20 to 25 repetitions. For rehabilitating athletes, MI of 4 weeks to 6 months duration, and lasting approximately 15 minutes, may be effective in reducing strength loss, increasing muscular activation, and decreasing negative psychological symptoms associated with injury (ie, pain perception, fear of reinjury, and injury anxiety).^7,36,78^ Such broad time frames suggest that MI is effective as both an acute and longer-term performance strategy.

## Factors to Consider When Using MI

It is important to recognize how skill level and sporting expertise, imagery ability, imagery perspective, and issues regarding instruction and amount of practice, may influence the effectiveness of MI.

### Individual Skill Level

An athlete’s skill level plays a significant role in the effectiveness of MI training. Previous research indicates that the benefits of MI are more pronounced in highly skilled athletes, with many years of training facilitating the development of intricate and detailed mental skill representations.^62,65^ In addition, it has been shown that the majority of elite athletes informally engage in MI, indicating they may have developed the capability to effectively implement more structured MI training.^16^ This suggests that the timescale for improvements may be shorter for high-level athletes, with novices requiring more time to develop the ability to vividly imagine and control their MI training. Consequently, we recommend explicit MI instruction in the early stages of MI training, and as the athlete’s capabilities improve, MI instruction may become less prescriptive, thereby providing the imager the opportunity for self-discovery.

### MI Ability

MI ability refers to an athlete’s capacity to create vivid and controllable mental images of their performance and maintain them long enough for effective visualization.^44^ Imagery ability relates to the degree to which an imager can guide their imagery experience. Improvements to imagery ability can result from imagery training,^5^ and athletes can be distinguished by imagery ability levels. Imagery ability is important as athletes with higher imagery abilities are likely to gain more from imagery training compared with those with lower abilities.^58^ These findings emphasize the importance of developing a vivid and controllable image to capture key performance components to elicit change.

### MI Perspective

MI perspective refers to the viewpoint the athlete adopts when imagining a movement. This perspective can be either internal (first-person) or external (third person). Both perspectives can be beneficial, but their efficacy may depend on the type of skill and the intended performance outcomes. For example, an external perspective could be appropriate for technique-focused movements (eg, gymnastic routine) as the athlete could imagine visual information regarding important technical information. If training is focused on developing an athlete’s physical qualities (eg, muscular strength), MI could aim to increase neurophysiological activity of the relevant muscles by using an internal perspective as this may enable the athlete to more accurately “feel” the imagined training.^76^

### Instructing MI

A crucial component of any MI training program is devising instructions on what and how the athlete will imagine in a given movement situation. As a way of facilitating the athlete to imagine the prescribed movement, MI scripts are commonly utilized. These scripts aim to present a detailed description of MI content to guide the athlete through the imagined training experience.^43^ A key recommendation in current MI practice is that scripts should be personalized and be developed in collaboration between the athlete and practitioner, allowing the athlete to determine MI content and the language used.^76^

**Figure 2.**
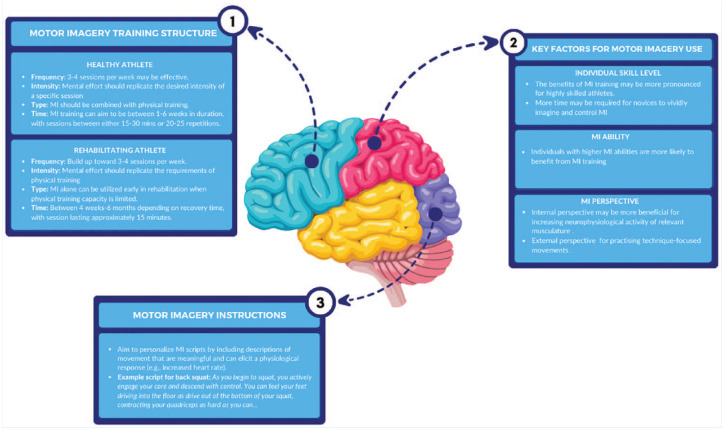
Key elements of MI training for load management. MI, motor imagery.

A robust strategy for developing personalized MI instructions is response training.^34^ Response training involves the athlete imagining themselves performing the movement of interest and recalling their experiences. Following this, the athlete’s responses to those imagined experiences are integrated into their MI script.^34^ For example, a basketball player may imagine themselves performing a lay-up during a training drill and recall experiencing “their leg muscles explosively extending as they jump into the shot.” This personally experienced response would be incorporated into MI, helping instructions to be meaningful to the athlete and potentially facilitating increased MI vividness. Figure 2 summarizes key elements of designing and delivering MI training.

## Limitations

MI is an established psychological training tool to help improve performance in sport; however, the role of MI as an additional training approach for the management of training load in sport has yet to be fully explored. The main aim of this article was to provide a narrative synthesis of the robust findings of MI to inform preliminary recommendations on the possible role of MI for load management. Drawing upon findings from a number of meta-analytic reviews and well-established frameworks for MI in sports performance,^37,53,54^ this review provides a foundation for future work implementing MI for load management. Although recommendations provided in this paper represent the starting point for the integration of MI as a load management strategy, a potential limitation of this work is that the idiosyncratic nature of MI, and the load management strategy employed, is influenced by dynamic contextual factors (eg, health of player, stage of season, team performance, injuries in the team, etc). As such, it is likely that these recommendations will need to be tailored to the individual organization to deliver the optimal outcome for the performer. Practitioners are advised to consider the teams’ training philosophy when integrating MI training as a load management strategy.

## Conclusion

The promotion of training load management has traditionally focused on recovery through complete rest^11^; however, this approach is limited as extended periods of rest have been associated with reduced local tissue and sport-specific capacities.^14^ Instead of total rest, MI provides a versatile cognitive strategy for managing training load by actively facilitating increased performance capabilities while also mitigating negative adaptations that may occur as a result of over- or undertraining. MI can also be applied to athletes across different stages of the training cycle. For example, during periods of high training load, MI can be used as a replacement, supplement, or alternative training strategy for physical training to promote recovery and maintain physical qualities, thus decreasing additional physical load and injury risk. For periods where training is reduced or completely removed, such as periods of injury or rehabilitation, MI alone could be considered to maintain some level of training without increasing the risk of injury and/or negative adaptations associated with prolonged periods of reduced training. Conversely, MI can be combined with physical training for healthy athletes to supplement physical training to elicit improved performance outcomes. The current guidelines offer sport medicine and performance staff an initial focal point for those wanting to utilize MI to effectively balance the training loads of their athletes and optimize time spent preparing for competition. Further research is necessary to better understand how to structure MI training for effective load management strategies.
